# Supplementary material for: Electrolyte Intake and Major Food Sources of Sodium, Potassium, Calcium and Magnesium among a Population in Western Austria
Source: Nutrients. 2020 Jun 30;12(7):1956. doi: 10.3390/nu12071956 (PMC7400604; doi:10.3390/nu12071956)
Supplement: Supplementary file 1 [file nutrients-12-01956-s001.pdf]

Supplementary Material

Table S1. Electrolyte intake in association with gender, age groups and dietary patterns. Data shown as mean values and standard deviation in parentheses in mg/day.

	**Sodium**	***P-Value***[1]	**Potassium**	***P-Value***	**Calcium**	***P-Value***	**Magnesium**	***P-Value***
women	2080.6 (821.7)		2713.7 (950.50)		869.6 (323.1)		318.1 (115.1)	
men	2981.4 (1248.9)		3161.9 (1243.6)		10945.0 (462.7)		389.5 (115.1)	
		<0.000		*<0.000*		*<0.000*		*<0.000*
age groups								
18 - 24 years	2697.7 (1252.9)		3142.1 (1136.6)		1119.9 (455.6)		388.5 (150.2)	
25 – 50 years	2573.7 (1144.0)		2960.1 (1189.3)		987.1 (409.7)		354.8 (137.6)	
51 – 64 years	2302.8 (1050.9)		2747.4 (937.0)		877.9 (366.3)		327.8 (106.1)	
		0.035		*0.050*		*<0.000*		*0.008*
women								
18 - 24 years	2102.3 (684.9)		2822.2 (924.7)		963.7 (282.1)		347.2 (125.4)	
25 – 50 years	2177.1 (902.6)		2666.7 (926.1)		866.0 (282.1)		311.1 (108.1)	
51 – 64 years	1853.2 (665.6)		2748.3 (1026.1)		817.3 (300.4)		315.2 (122.2)	
		0.037		*0.633*		*0.085*		*0.220*
men								
18 - 24 years	3342.7 (1409.6)		3488.6 (1251.2)		1289.1 (543.9)		433.3 (163.2)	
25 – 50 years	2973.4 (1222.9)		3255.7 (1345.5)		1109.2 (438.2)		398.7 (149.9)	
51 – 64 years	2775.7 (1173.0)		2746.3 (842.1)		941.6 (418.0)		341.0 (84.9)	
		0.100		*0.007*		*0.001*		*0.004*
Dietary patterns								
Health conscious	1999.7 (993.6)		3176.6 (1438.2)		911,2 (343.4)		391.9 (171.4)	
Traditional	2592.6 (1064.2)		2685.8 (796.1)		961.3 (405.6)		320.9 (96.0)	
Western	3358.5 (1346.4)		3834.4 (1369.0)		1257.9 (499.0)		453.1 (150.4)	
		<0.000		*<0.000*		*<0.000*		*<0.000*

[1] *P*-Values for group differences are based on ANOVA for metric variables

Table S2. Percentage of persons with a discrepancy between reported intake and the recommended values for sodium, potassium, calcium and magnesium in different age groups.

	lower intake	adequate intake	higher intake
**Sodium (Na)**			
	(< 1350 mg/day)	(1350 - 1650 mg/day)	(> 1650 mg/day)
18-24 years, n=75	12.0 %	5.3 %	82.7 %
25-50 years, n=269	10.8 %	6.3 %	82.9 %
51-64 years, n=119	21.0 %	3.4 %	75.6 %
**Potassium (K)**	lower intake	adequate intake	higher intake
	(< 3800 mg/day)	(3800 – 4200 mg/day)	(> 4200 mg/day)
18-24 years, n=75	74.7 %	10.7 %	14.7 %
25-50 years, n=269	84.4 %	6.3 %	9.3 %
51-64 years, n=119	89.9 %	5.0 %	5.0 %
**Calcium (Ca)**	lower intake	adequate intake	higher intake
	(< 950 mg/day)	(950 – 1050 mg/day)	(> 1050 mg/day)
18-24 years, n=75	42.7 %	10.7 %	46.7 %
25-50 years, n=269	52.0 %	6.7 %	41.3 %
51-64 years, n=119	58.8 %	14.3 %	26.9 %
**Magnesium (Mg)**	lower intake	adequate intake	higher intake
	(< 285 mg/day)	(285-420 mg/day)	(> 420 mg/day)
18-24 years, n=75	29.3 %	40.0 %	30.7 %
25-50 years, n=269	30.5 %	45.4 %	24.2 %
51-64 years, n=119	40.3 %	44.5 %	15.1 %
